# Cancer pain self-management interventions in adults: scoping review

**DOI:** 10.1136/spcare-2024-004893

**Published:** 2024-05-07

**Authors:** Elly L Sjattar, Rosyidah Arafat, Lee Wan Ling

**Affiliations:** 1 Hasanuddin University, Makassar, Indonesia; 2 University of Malaya, Kuala Lumpur, Malaysia

**Keywords:** Pain

## Abstract

**ABSTRACT:**

**Background:**

The predominant trend in cancer treatment now leans towards outpatient care, placing the responsibility of pain management largely on the patients themselves. Moreover, a significant portion of treatment for advanced cancer occurs in the home environment, so patient self-management becomes increasingly crucial for the effective treatment of cancer pain.

**Objectives:**

To map self-management for pain in patients with cancer at all phases of the disease before examining the potential of pain self-care interventions for ill patients with cancer.

**Methods:**

A search was conducted on six electronic databases to locate studies published in English, from 2013 to 2023. We followed Arskey and O’Malley’s Scoping Reviews guidelines.

**Results:**

This study thoroughly examined the provision of cancer pain self-management by healthcare professionals and identified four intervention types from 23 studies. Education emerged as the most prevalent form of self-management for cancer pain.

**Conclusion:**

*G*uiding patients in managing their pain effectively, starting from their hospitalisation and extending to their discharge.

**WHAT WAS ALREADY KNOWN?**

- Self-management increasingly crucial for effective treatment of cancer pain.
- In outpatient care, pain management largely on the patients themselves.

**WHAT ARE THE NEW FINDINGS?**

- The mapping of cancer pain self-management interventions.
- Cancer pain-self care through educational programmes.

**WHAT IS THEIR SIGNIFICANCE?**

a. Clinical: Nurses should support patients with cancer to attain pain self-management.

b. Research: More definitive trials required to determine pain-self care efficacy.

## Introduction

Pain is a predominant symptom frequently experienced by individuals diagnosed with cancer.^1^ The prevalence of cancer pain is approximately 54.6%, especially among persons with advanced, metastatic and terminal stages.^2^ Unresolved pain in patients with cancer causes disturbances in comfort, capability, motivation and interactions with family and friends, as well as reducing quality of life. This contributes to the onset of depression, anxiety, life dissatisfaction, sexual difficulties and difficulties in carrying out everyday tasks. Unmanaged pain significantly increases the likelihood of patients contemplating suicide.^3 4^

Various strategies, both pharmaceutical and non-pharmaceutical, have been used to alleviate pain. Among the non-pharmaceutical approach to pain management includes methods such as massage, acupressure, specific exercises, temperature modulation, and cognitive-behavioural techniques like mindfulness and relaxation. Additionally, psychosocial support and spiritual guidance are offered to patients in medical facilities or hospice settings.^5^ The predominant trend in cancer treatment now leans towards outpatient care, placing the responsibility of pain management largely on the patients themselves. Moreover, a significant portion of treatment for advanced cancer occurs in the home environment. As a result, patient self-management becomes increasingly crucial for the effective treatment of cancer pain.^6 7^

Self-management of cancer pain refers to measures or actions undertaken by affected individuals to reduce pain symptoms and to cope with the physical, emotional and social consequences and lifestyle changes caused by chronic pain. They improve their self-efficacy by tackling issues associated with pain and integrate strategies for pain relief into their everyday lives through engagement with healthcare professionals. Self-management is recommended to improve cancer pain awareness, self-management behaviours and adaptive abilities of patients.^6 8^ Self-management of pain often involves combination of strategies and the guidance available for patients with cancer to manage their pain autonomously is still limited. This scoping review sought to map the self-management interventions or modalities of cancer pain management before examining the potential of self-care interventions in enhancing health outcomes.

## Methods

This scoping review was guided by the methodological framework described by Arksey and O’Malley. The five stages undertaken were: (i) identifying the research question; (ii) identifying the relevant studies; (iii) study selection; (iv) charting data and (v) collating, summarising and reporting results.^9^ Reporting of scoping review adhered to guidelines of the Preferred Reporting Items for Systematic Reviews and Meta-Analyses (PRISMA) extended version for Scoping Reviews (PRISMA-ScR).^10^

### Identifying the research question

A comprehensive review of existing literature was undertaken to explore methods of self-management for patients with cancer dealing with pain. The research question for this study was ‘What types of self-management are available to alleviate cancer pain?’

### Identifying the relevant studies

This comprehensive scoping review included a broad range of sources, covering both quantitative and qualitative research studies from a variety of databases: Medline, Clinical Key, Cochrane, Sciences Direct and Proquest were searched from 2013 to April 2023 to identify current management in the last 10 years. The terms and formulas used for conducting the search are outlined as follows: (“self management” OR “Self care”) AND pain AND (Cancer or tumour or oncology or neoplasm). Studies included in the review must met criteria for research purposes. The inclusion criteria were studies that reported: self-management interventions, studies with qualitative and quantitative designs were included, all types of cancer. Literature reviews and protocols written in languages other than Indonesian and English were excluded.

### Study selections

**Figure 1:**
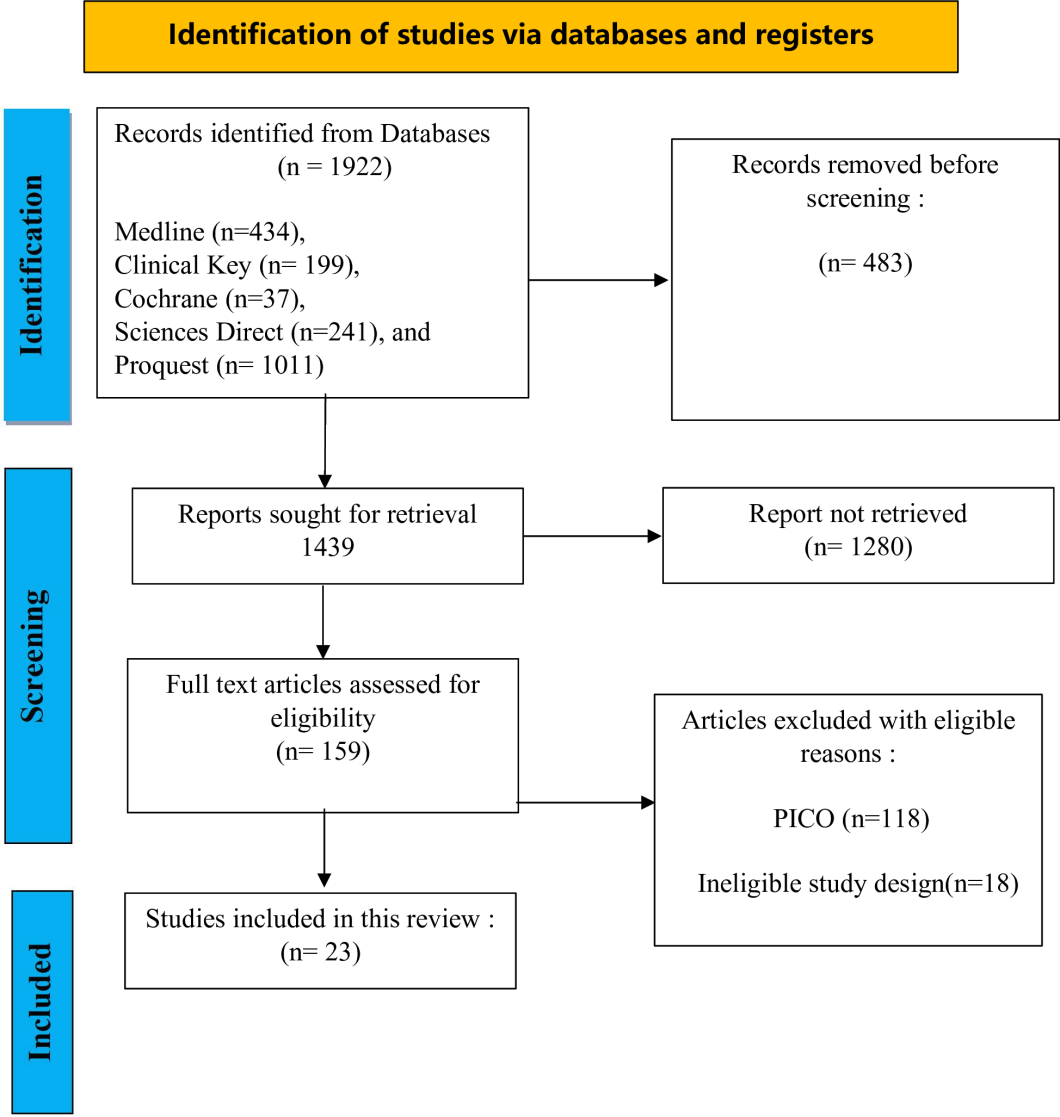
PRISMA Sc-R Flow Diagram, adapted from Tricco et al.^10^

Two researchers (ES and RA) independently reviewed the titles and abstracts of all studies, followed by screening the complete studies against the eligibility criteria. Any discrepancies in study selection were resolved through discussion. A detailed description of the process for screening and article selection can be found in figure 1.

### Charting data

A structured form was derived to extract information on study characteristics, such as the primary author’s name, publication year, country of study conducted, study aim and design, samples and sampling (which includes sample size, sampling criteria and method, sample characteristics, types and phases of cancer), the self-management interventions including providers of intervention, measurement of outcomes and its instruments used, and the findings on the interventions. Both researchers independently extracted the data using this form.

### Collating, summarising and reporting results

The data extracted from both researchers were compared and summarised into a single table for analysis. The findings were reported in tables with detailed descriptions of study characteristics and self-management interventions to alleviate cancer pain. The scoping review is reported according to the PRISMA-ScR checklist.^10^

## Results

### Characteristics of studies

This review examines the latest self-management of cancer pain either in the community or hospital settings in the last 10 years. It includes patients’ self-care practices and coping skills when they were undergoing chemotherapy and radiotherapy. A total of 23 studies conducted from 2013 to 2023 were reviewed. The majority of studies were from Western countries, that is, USA (n=3), Australia (n=2), Netherlands (n=2), Switzerland (n=2), Germany (n=2), Austria (n=1), Norway (n=1), Canada (n=1). Studies conducted in Eastern-cultured countries are as follows: China (n=3), Iran (n=2), Japan (n=1), Vietnam (n=1), Kenya (n=1), Indonesia(n=1), Turkey (n=1). Online supplemental table 1 presents a summary of quantitative (n=15) and mixed method studies (n=5), while online supplemental table 2 on qualitative studies (n=3).^11^,^32^

### Study design

The experimental designs were the most common approach employed in quantitative studies, that is, randomised controlled trials (n=9), prospective non-randomised trial (n=1), multicentre pilot clinical study (n=2), feasibility studies (n=1), prospective case–control study (n=1), survey (n=1) and mix methods (n=5). These designs mainly aim to examine the effects of interventions. The common designs for the qualitative studies were phenomenology (n=1), grounded theory (n=1), case study approach with in-depth interview (n=1), which were mainly employed to inform development of interventions.^11^,^32^

### Participant characteristics

Sample sizes in the quantitative and mixed methods studies varied from a minimum of 16^11^ to largest sample of 688 participants.^12^ In the qualitative studies, sample sizes ranged from a minimum of 8^13^ to 22 participants.^14^ While some studies target on participants with one type of cancer (eg, bone, breast, oral), most of the studies includes participants of various cancer type.

### Pain and other measurements of outcomes

The Numeric Rating Scale^1112 15^,^25^ was the most widely used tool to measure cancer pain. Other pain measures include Brief Pain Inventory^26^ and its Short Form,^27 28^ Visual Analogue Scale and American Pain Society Patient Outcome Questionnaire.^29^ While cancer pain is the primary measure, other outcomes measures include self efficacy,^17 18 23^ quality of life,^12 22 23 29^ patient empowerment,^12^ medication adherence,^16 19^ disability,^27^ anxiety and depression.^23^

### Characteristics of self-management interventions for cancer pain

The interventions were mostly delivered by nurses,^1115^,^21 24 28^ while some studies had multi-disciplinary teams that includes nurses, clinical psychologists, doctors, counsellors, pharmacists, social workers, healthcare professionals and the research team.

As shown in online supplemental table 3, most studies deliver interventions to combination of individual and group approach. Techniques for providing education also vary, whether provided directly or indirectly (telephone, apps and web). The educational process is conducted through home visits^15 24 25^ and telephone calls to provide guidance on pain management,^12 15 18 20 21 24 25 30^ with some using platforms like Microsoft Teams, Zoom or WhatsApp.^26^ Sessions can last up to 60 min, and there is also the development of a self-management resource model targeting various barriers.^31^ Other intervention programmes, such as the Self Care Improvement through Oncology Nursing-PAIN programme, have shown promise in reducing patient barriers and improving compliance with pain treatment.^19^ Additionally, models like the ‘Holistic Supporting from Pain Self-Management’ offer insights into factors supporting pain self-management.^14^ Through the Delphi technique and patient trials, the Cancer Pain Belief Modification Programme emerges as a potential tool for managing oral cancer pain.^22^

The intervention periods ranged from 24 days^19^ to 11 months.^12^ For interventions lasting 4 months, monitoring for 4 weeks is necessary.^16^ The duration for conducting pain management education spans 7 weeks, with additional assessments at 1 and 3 months postintervention.^17 29^ Alternatively, it can extend for 8 weeks, starting a week before the patient’s hospital discharge until a week after returning home.^28 32^

## Discussion

Based on the mapping results of 23 studies in this review, there are four forms of integration of education with pain self-management, including conducting interventions through face-to-face interactions and online platforms (phone, video conferencing, eHealth, Microsoft Teams, Zoom or WhatsApp); creating models, guidebooks and questionnaires; developing and modifying programmes or interventions for pain self-management tailored to cancer/patient types; and non-pharmacological pain management such as peripheral therapy (hot and cold compresses, transcutaneous electrical nerve stimulation, acupuncture, acupressure, massage, hydrotherapy, exercise) and cognitive behavioural therapy (relaxation techniques, meditation, prayer, yoga, hypnosis, biofeedback).^911 12 16 18 21 27^,^29 32^

All intervention in this review emphasises the self-pain management process of patients with cancer, where pain self-care is an action taken by a person to be able to control cancer pain in their daily life. In line with various aspects of self-care for managing pain, including: engaging with healthcare providers, making decisions about pain management, problem-solving related to pain, building self-confidence and integrating different strategies for alleviating pain in daily activities.^8 33^

Primarily, findings from this research indicate that interactions with healthcare professionals occur predominantly through educational programmes, delivered in both face-to-face and online formats. These programmes aim to provide comprehensive information on diverse pain reduction methods, empowering patients to make informed decisions about managing their pain effectively. Efforts to address cancer-related pain should aim to improve patients’ comprehension and skills, promoting an active approach to pain management. This involves providing education about pain characteristics, treatment choices, their impacts and integrating non-pharmacological methods.^34^,^36^

Nurses should offer support and feedback to encourage individuals to attain self-management, leading to an increased awareness of enhanced self-efficacy. This helps establish a routine of applying pain relief knowledge and skills in daily life, adapting to fluctuations in pain levels.^8^ Achieving this necessitates prolonged monitoring, with the duration of this comprehensive self-management programme ranging from 3 weeks to 11 months.

The limitations of this scoping review are associated with the search methodologies and the criteria used for inclusion. The review only considered English-language articles, which could lead to bias and hinder the comprehensiveness of the study. Additionally, all the data gathered in this study can support healthcare professionals, especially nurses, in providing thorough care to patients with cancer. The emphasis is on guiding patients in managing their pain effectively, starting from their hospitalisation and extending to their discharge. This intensive approach is geared towards achieving pain alleviation, improving overall well-being, and fostering empowerment.

## Conclusion

This study examined the provision of self-management intervention by healthcare professionals to address patients’ cancer pain. Among the four intervention types identified from 23 studies, educational intervention emerged as the most popular approach. Delivery of educational intervention includes face-to-face and online mode. Other common approaches included non-pharmacological pain management techniques and cognitive-behavioural therapy.

## supplementary material

10.1136/spcare-2024-004893 online supplemental file 1

## Data Availability

The information related to this review is displayed within the manuscript, figures, and tables (Supplementary tables).
