# Supplementary material for: Cancer pain self-management interventions in adults: scoping review
Source: BMJ Support Palliat Care. 2024 May 7;14(4):e004893. doi: 10.1136/spcare-2024-004893 (PMC11671981; doi:10.1136/spcare-2024-004893)
Supplement: online supplemental file 1 [file spcare-14-4-s001.pdf]

Table 1. Articles Exploring Pain Self-Management for Cancer (Quantitative and Mixed Method Studies)

No	Author<br>Year<br>Country	Study aim<br>Study design	Population (includes types & phases of cancer)<br>Study setting<br>Sample size	Interventions descriptions (including duration, providers)	Pimary Outcome measures<br>Instruments	Primary outcomes and key findings
1	Rustoen, et al (2014)<br><br>Norwegia	To evaluate the effectiveness of the modified PRO-SELF Pain Control Program for Norwegian cancer patients in reducing pain and increasing opioid intake compared with control treatments<br><br>(Randomized Controlled Trial/RCT)	Bone cancer<br><br>Metastatic bone cancer<br><br>Intervention Group (n=87) & control group (n=92)<br><br>In Oncology Outpatient	• Nurses visit the patient's home at weeks 1, 3, and 6 to provide education about the pain they are feeling.<br>• Nurses contacted patients by telephone and reviewed pain intensity scores and analgesic intake at weeks 2, 4, and 5.	Numeric Rating Scale (NRS)	• In both the intervention and control groups, a decrease in pain intensity scores was found (both p<0.001) over studies. However, no significant time-group interaction was found for the given pain intervention.<br>• In both groups, the total opioid dosage taken increased over time. However, a non-significant time-group interaction was found for changes over time in total dosage, cumulative dosage, or as-needed opioid analgesic dosage.
2	Kelleher, et al (2021)<br><br>USA (North and south Carolina, Georgia)	• Testing the extent to which mPCST Community reduces levels of pain, pain interference, fatigue, physical disability, and psychological distress in breast	Breast Cancer<br><br>Breast Cancer (stadium 0-IV)<br><br>180 sample<br><br>At medical center	The intervention is conducted via video conferencing for 50 minutes and a mobile application divided into 4 sessions.<br>• Session 1 focuses on the experiences of cancer patients and provides a brief overview of age-specific cancer pain	Brief Pain Inventory-Short Form (BPI-SF).	The mPCST-Community has the potential to reduce pain and disability, as well as lower barriers for breast cancer patients in medically underserved areas.

No	Author<br>Year<br>Country	Study aim<br>Study design	Population (includes types & phases of cancer)<br>Study setting<br>Sample size	Interventions descriptions (including duration, providers)	Pimary Outcome measures<br>Instruments	Primary outcomes and key findings
		cancer patients.<br>● Examining potential mediators (such as self-efficacy, pain coping) of the effects of mPSCT-Community.<br><br>● Evaluating mPSCT-Community and cost-effectiveness.<br><br>(Randomized Controlled Trial/RCT)		information (prevalence).<br>● Session 2 focuses on general medical care as well as its benefits and side effects.<br>● Session 3 includes information on communicating with the healthcare team.<br>● Session 4 provides basic information on healthy eating patterns and activity levels.		
3	Lovell, et al (2022)<br><br>Australia	To measure whether the intervention yields a 30% increase in pain scores among adults attending outpatient oncology or palliative care clinics with a Numeric Rating Scale (NRS) score of 5 or higher.<br><br>(Cluster-Randomized trial)	All cancer<br><br>Intervention Group (n=329) & control group (n=359)<br><br>At oncology and palliative care outpatient clinics	The strategy of implementing guidelines at the cluster level, healthcare professionals, and patients is introduced with the support of clinical leaders.	Numeric Rating Scale (NRS)	For the primary outcome, the proportion of participants with a decrease in pain scores of 5 or more by 30% in the control and intervention phases (31 out of 280 participants [11.9%] vs 30 out of 264 participants [11.8%]; OR, 1.12; 95% CI, 0.79-1.60; P = .51). No significant difference was found in the secondary outcomes between the two phases.
4	Son Dinh Vu, et al (2023)	To evaluate the effectiveness of pain management support	All cancer<br><br>116 patient with cancer pain	The intervention is conducted through health education consultations. The intervention	The Brief Pain Inventory- Short Form (BPI-SF)	In the intervention group, there were statistically significant differences in the mean scores before intervention

No	Author<br>Year<br>Country	Study aim<br>Study design	Population (includes types & phases of cancer)<br>Study setting<br>Sample size	Interventions descriptions (including duration, providers)	Pimary Outcome measures Instruments	Primary outcomes and key findings
	Vietnam	intervention on pain and its interference with daily activities in cancer patients in Vinh Phuc Province after 1 week of discharge from the hospital.<br><br>(Randomized Controlled Trial/RCT)	At Vinh Phuc Provincial General Hospital (Before patients leaves hospital)	content includes providing information about pain, building pain management skills, and supporting cancer patients with self-care.		and 1 week after discharge from the hospital for the most severe pain (4.88 ± 1.55 and 3.64 ± 1.45, p <0.05), least severe pain (2.14 ± 1.05 and 1.68 ± 0.92, p <0.05), moderate pain (3.54 ± 1.39 and 2.38 ± 1.04, p <0.05), current pain (3.09 ± 1.76 and 2.25 ± 1.25, p <0.05), overall pain (3.41 ± 1.28 and 2.49 ± 1.07, p <0.05), and pain interference with daily activities (4.23 ± 1.75 and 3.48 ± 1.91, p <0.05). The study also showed that there were statistically significant differences in mean scores between the intervention group and the control group at 1 week after discharge from the hospital for the most severe pain (3.64 ± 1.45 and 5.00 ± 1.71, p <0.05), moderate pain (2.38 ± 1.04 and 3.46 ± 1.61, p <0.05), current pain (2.25 ± 1.25 and 3.31 ± 1.88, p <0.05), overall pain (2.49 ± 1.07 and 3.50 ± 1.52, p <0.05), and pain interference with daily activities (3.48 ± 1.91 and 4.35 ± 2.18, p <0.05). The effect size coefficient for overall pain intensity was moderate (Cohen's d = 0.76), and the effect size coefficient for pain interference with daily activities was small

No	Author<br>Year<br>Country	Study aim<br>Study design	Population (includes types & phases of cancer)<br>Study setting<br>Sample size	Interventions descriptions (including duration, providers)	Pimary Outcome measures<br>Instruments	Primary outcomes and key findings
						(Cohen's d = 0.42).
5	Yang, et al (2022)<br><br>China	To evaluate the impact of whole process management intervention based on the benefits of the information system reported by cancer pain patients.<br><br>(Prospective Non-Randomized Controlled)	All cancer<br><br>124 patients with cancer pain<br><br>At the hospital and continue to out hospital	Patients in the intervention group receive the entire process management model intervention based on the information system compared to the control group, which receives routine cancer pain management.	Numeric Rating Scale (NRS)	The primary outcome is the change in four aspects of pain management quality (pain management quality, Patient-related attitudinal barriers, Analgesic adherence) (APS-POQ-C) between the two groups (P <0.05). Patients in the intervention group reported much better pain control and service perception compared to the control group. Regarding secondary endpoints, significant differences in support for the experimental group were found for barriers (P <0.05) and medication adherence (60.0% vs 40.0%; P <0.05) after the intervention.
6	Valenta, et al (2021)<br><br>Switzerland	To explore the learning process related to psychoeducational interventions for pain management.<br><br>(Mix-methods)	All cancer<br><br>21 patients and 7 caregiver<br><br>Outpatient	The intervention consists of education provision, skill development, and nursing coaching.	Numeric Rating Scale (NRS)	Both groups showed an increase in knowledge (p = 0.035) and self-efficacy (p = 0.007). Qualitative data indicate three themes corresponding to intervention components: Experiencing uncontrolled pain and side effects, Learning from competent and trustworthy coaches during the intervention, and Experiencing success in enhancing pain management strategies.

No	Author Year Country	Study aim Study design	Population (includes types & phases of cancer) Study setting Sample size	Interventions descriptions (including duration, providers)	Pimary Outcome measures Instruments	Primary outcomes and key findings
7	Hochstenbach, et al (2016)<br><br>The Netherlands	This study describes the feasibility of the intervention in everyday practice.<br><br>(One group post test only design/Feasibility study)	13 patients with cancer pain and 3 specialist nurse in palliative.<br>All cancer<br>Palliative care	This intervention involves daily monitoring, graphical feedback, education, and advice via a mobile application for patients and a web application for nurses.	Numeric Rating Scale (NRS)	Based on the research findings, the questionnaire components are learning ability (4.8), usefulness (4.8), and desirability (4.6) of the application for patients. The average completion rates are 76.8% for pain monitoring, 50.4% for medication monitoring, and 100% for educational sessions. Interview results indicate that patients are pleased with the simplicity of the mobile application and appreciate its various components. Nurses agree on the added value and mostly have a positive attitude toward the use of the web application.
8	Raphaelis, et al (2020)<br><br>Austria/Vienna	To report the evaluation of the implementation of the ANtiPain self-management intervention in a realistic German-language environment in the domains of Reach, Effectiveness, and Implementation.<br><br>(Cluster-Randomized trial)	All cancer<br><br>157 patients with cancer pain<br><br>At the hospital	Patients receive appropriate leaflets, individual treatment plans, and pain scale figures. Nurses adhere to the intervention protocol, apply assessment instruments (for example, to assess pain or barriers in pain management), and are asked to use a pocketbook on common analgesics.	Numeric Rating Scale (NRS) dan The Brief Pain Inventory (BPI)	Face-to-face sessions (average duration = 33 minutes) as well as the average number (n = 2) and duration of telephone calls (average = 17 minutes). Only 16 (46%) out of 35 trained nurses conducted interventions in nine wards. The overall effect on pain interference was not significant. However, the effect was significant in the sub-analysis of the nine wards recruiting patients during the intervention period ($p=0.009$). Regarding secondary outcomes, there was a

No	Author<br>Year<br>Country	Study aim<br>Study design	Population (includes types & phases of cancer)<br>Study setting<br>Sample size	Interventions descriptions (including duration, providers)	Pimary Outcome measures<br>Instruments	Primary outcomes and key findings
						significant group-by-time effect on self-efficacy (p = 0.033), and patient satisfaction with self-management pain information (p= 0.002) and pain management in the hospital (p = .018).
9	Jahn, et al (2014)<br><br>Germany	To test the Self-Care Improvement in Oncology Nursing (SCION)-PAIN program, a structured multimodal intervention aimed at reducing patient barriers in managing cancer pain independently.<br><br>(Cluster-Randomized trial)	All cancer<br><br>263 cancer patients<br><br>At oncoligical ward	The intervention components consist of patient education, skills training, and counseling. Starting upon admission to the hospital, patients receive booster sessions every third day and one session of follow-up telephone counseling within 2 to 3 days after discharge from the hospital. Patients in the control group receive standard care.	Numeric Rating Scale (NRS	The SCION-PAIN program resulted in a significant reduction in patient barriers to pain management 1 week after discharge from the hospital: the mean difference on the BQ II was 0.49 points (95% confidence interval 0.87 points to 0.12 points; P=0.02). Furthermore, patients showed improvement in adherence to pain medication; odds ratio 8.58 (95% confidence interval 1.66–44.40; P = 0.02).
10	Jibb, et al (2016)<br><br>Canada	To evaluate the feasibility of implementing the Pain Squad+ application as the intervention group in a future randomized trial. The second objective is to obtain estimates of the treatment's impact on adolescent health outcomes.<br><br>(Multocenter pilot	All cancer<br>2 months since diagnosis<br>40 samples<br><br>At the hospital	Intervention recommendations may be pharmacological (e.g., reminders to adhere to prescribed medication), psychological (e.g., distraction techniques), and/or physical (e.g., yoga instructions).	Numeric Rating Scale (NRS	Intervention fidelity is influenced by technical difficulties (occurred in 15% of participants) and the time taken to contact a nurse in case of sustained pain. Compliance with pain reporting is 68.8 ± 38.1%. Completion rates of outcome measurements are high, and the intervention is acceptable to participants. There are significant trends in increasing pain intensity, pain interference, and HRQL, with effect sizes of 0.23–0.67.

No	Author Year Country	Study aim Study design	Population (includes types & phases of cancer) Study setting Sample size	Interventions descriptions (including duration, providers)	Pimary Outcome measures Instruments	Primary outcomes and key findings
		clinical study)				
11	Koller, et al (2017)<br><br>Germany	To assess the effects of ANtiPain on pain intensity, function-related outcomes, self-efficacy, and patient-related barriers in pain management to prepare for a larger effectiveness trial.<br>(Pilot-RCT)	All cancer<br><br>Intervention Group (n=20) & control group (n=19)<br><br>At cancer center	The ANtiPain intervention includes structured and tailored components and is based on 3 main strategies: information, skill development, and nursing coaching.	Numeric Rating Scale (NRS)	Large effects were found for activity interference (Cohen's d=0.90), interference (d=0.91), and self-efficacy (d=0.90). Small to moderate effects were found for moderate and worst pain (Cohen's d=0.17-0.45).
12	Wang, et al (2023)<br><br>China	To develop a cancer pain management program based on belief modification to enhance the pain experience of oral cancer survivors in China and to explore the acceptance and initial outcomes of the Cancer Pain Belief Modification Program (CPBMP).<br><br>(Mix-methods)	Oral cancer<br><br>16 patients with oral cancer<br><br>At the hospital	-	Numeric Rating Scale (NRS)	"Intense pain", "negative pain beliefs" scores before and after testing decrease[illegible] from 5.63±0.48 to 0.81±0.54 (t=-3.746[illegible] p<0.001); from 140.63±9.02 to 52.75±[illegible] (Z=12.406, p<0.001); and scores of "positive pain beliefs", "quality of life[illegible] increased from 55.13±4.54 to 66.00±4.[illegible] (Z=-6.983, p<0.001); from 66.97±15.0[illegible] 86.69±8.42 (Z=7.283, p<0.001). Qualitative data also showed that CPB[illegible] was well accepted.
13	Musavi, et al (2021)<br><br>Iran	To determine the impact of pain management education on the severity of pain and quality of life in metastatic cancer patients using a complementary treatment	All cancer<br>Metastatic cancer<br>75 cancer patients with metastatic<br><br>At Oncology clinic	Pain management is taught in three steps: providing information, skill development, and guidance.	the Visual Analog Scale (VAS)	After the intervention, the severity of pain during weeks 1-7 differed significantly between the intervention and control groups (P <0.0001). Additionally, significant differences were observed in quality of life at 1 and 3 months after the

No	Author<br>Year<br>Country	Study aim<br>Study design	Population (includes types & phases of cancer)<br>Study setting<br>Sample size	Interventions descriptions (including duration, providers)	Pimary Outcome measures<br>Instruments	Primary outcomes and key findings
		approach.<br><br>(RCT)				intervention between the two groups studied (P <0.0001).
14	Bilmiç, et al (2023)<br><br>Turkiye	To evaluate the effectiveness of an individual online education program on patient-related barriers in cancer pain management.<br><br>(RCT)	All cancer<br>110 participants in this study<br><br>At outpatient chemotherapy	Individual online education is conducted as the intervention, provided in addition to standard care; delivered through printed educational materials and in an online format.	Brief Pain Inventory (BPI)	Both groups (F = 11.316, p = 0.001) and time effect (F = 63.878, p <0.001) individually have a significant influence on the total BQII score. Additionally, there are significant differences between groups regarding the total BQII score regardless of time. The interaction between group and time is also significant (F = 127.764, p <0.001) and substantially affects the total BQII score. Regarding pain intensity, the results indicate that the interaction between group and time is statistically significant for all pain categories (p <0.05). Conversely, the group effect is not statistically significant for all pain categories (p > 0.05). The time effect is statistically significant only for "least pain" and "average pain" (p <0.05).
15	Xin Yin, et al (2023)<br><br>China	To improve symptoms of chronic pain, as well as anxiety and depression related to cancer through the PRO-SELF nursing	All cancer<br>Diagnosed phase<br><br>Intervention Group (n=34) & control group (n=30)	The control group receives routine cancer pain nursing interventions, while the intervention group receives a collaborative multidisciplinary	Numeric Rating Scale (NRS)	No significant differences were found between the groups in terms of pain and medication adherence (P > 0.05). However, significant differences were found between the

No	Author Year Country	Study aim Study design	Population (includes types & phases of cancer) Study setting Sample size	Interventions descriptions (including duration, providers)	Pimary Outcome measures Instruments	Primary outcomes and key findings
		intervention.<br><br>(Prospective case control study)	At outpatient departement	PRO-SELF-based cancer pain nursing intervention.		groups in social support, quality of life, self-efficacy for chronic pain, and self-assessment of anxiety and depression index scores (P <0.001). The intervention group reported more social support, self-efficacy for pain, and less anxiety and depression (P <0.001).
16	Hochstenbach, et al (2017)<br><br>The Netherlands	This study describes a creative method aimed at developing eHealth interventions delivered by registered nurses to support self-management in outpatient cancer pain patients.<br><br>(Mix-methods)	Squamous cell carcinoma, breast cancer, prostate cancer, and neurosarcoma<br><br>5 sample with cancer pain<br><br>in outpatient	The development process resulted in interventions in the form of home visits, a mobile application for patients, a web application for caregivers, and follow-up activities.	-	Development Process : Content exploration (1-3 months), content spesification (4-5 months), care organization (6-9 months).<br><br>Intervention Content : Home visit, mobile application for patients, web application for nurses, follow-up activities by nurses
17	Luckett, et al (2019)<br><br>Australia	To report on the stages of development, feasibility, and testing for patient self-management resources.<br><br>(Mix-methods)	-	Systematic review of cancer pain needs and education; online desktop review of patient pain diaries and other related resources; consultation with stakeholders; and interviews with patients regarding their acceptance.	-	Optimal self-management resources can encourage reporting of perceived pain, build patient control, and support communication with service providers and service coordination. Pain management resources developed include: (1) templates for setting specific, measurable, achievable, relevant, and time-bound service goals, as well as assessing potential barriers and ways to overcome them. (2) pain management plans detailing factors

No	Author<br>Year<br>Country	Study aim<br>Study design	Population (includes types & phases of cancer)<br>Study setting<br>Sample size	Interventions descriptions (including duration, providers)	Pimary Outcome measures<br>Instruments	Primary outcomes and key findings
						that can exacerbate and alleviate pain, current management strategies, and contacts for support.
18	Yamanaka, Masako & Suzuki, Kumi (2020)<br><br>Japan	For the development of a nursing intervention program designed to promote self-management of pain for adult outpatient cancer patients to address cancer pain-related issues and evaluate the suitability and clinical implementation of the program.<br><br>(Survey)	2 pharmacists and 10 oncology specialist nurses<br><br>-	-	-	The average score for all 23 items is 4.3 (standard deviation [SD] 0.6). The average item scores are 4.4 (SD 0.6) for program suitability, 4.2 (SD 0.5) for clinical program implementation, and 4.3 (SD 0.7) for the suitability of instructional materials.
19	Valenta, et al (2022)<br><br>Switzerland	To evaluate the effectiveness of pain management intervention compared to standard care and to explore participants' experiences in pain management and research participation.<br><br>(RCT)	All cancer<br>21 cancer patients & 7 caregivers<br><br>Outpatient	The intervention group received a six-week intervention consisting of education, skill development, and nurse coaching. The control group received standard care.	The Brief Pain Inventory (BPI) dan Numeric Rating Scale (NRS	The effect showed a statistically significant decrease in average pain ($P = 0.04$), but there was no significant time x group effect for worst pain ($P = 0.06$). Pain scores, pain-related knowledge, Pain Management Index, self-efficacy, and performance status improved in the intervention group ($P < 0.05$).
20	Schumacher, et al (2019)<br><br>Northern	To longitudinally describe patients' pain experiences over a 10-	All cancer<br>42 patients<br><br>Oncology Outpatient	Psychoeducation Pain Management Intervention	Numeric Rating Scale (NRS)	This analysis reveals the remarkable dynamic nature of each patient's pain experience. Various aspects of pain contribute to its dynamic nature,

No	Author<br>Year<br>Country	Study aim<br>Study design	Population (includes types & phases of cancer)<br>Study setting<br>Sample size	Interventions descriptions (including duration, providers)	Pimary Outcome measures<br>Instruments	Primary outcomes and key findings
	California	week pain management intervention.<br><br>(Mix-methods)				including fluctuating pain locations, concurrent emergence of pain sources, and varying patterns of pain intensity over time. For each patient, the cumulative effect of these various aspects results in a phenomenon referred to as "complex pain dynamics."

Tabel 2. Articles Exploring Pain Self-Management for Cancer (Qualitative Studies)

No	Author<br>Year<br>Country	Study aim<br>Study design	Population (includes types & phases of cancer)<br>Study setting<br>Sample size	Primary outcomes and key findings
21	Hassankhani, et al (2023)<br><br>Iran	For the development of a supportive model to enhance self-management of cancer pain based on the grounded theory study "doubtful persistent effort to pain relief".<br><br>(Grounded Theory)	All cancer<br><br>22 sample : 17 patients, 2 caregiver, 2 nurses, and 1 docter<br><br>At the hospital	Based on the qualitative analysis, 14 sub-categories were identified within 4 main categories. The sub-categories include (sensory perception of pain, pain generators, internal self-control, freeing oneself from spontaneous negative thoughts, traditional approach influenced by other approaches, medical care, accepting and enduring pain, negative attitudes towards analgesic effectiveness, low patient knowledge about pain management methods, healthcare providers as barriers, institutional barriers, mood disturbances, feelings of worthlessness, and family chaos).
22	Makena, et al (2023)<br><br>Kenya	To evaluate nursing interventions in promoting self-management of cancer pain at Tenwek Hospital, Bomet County, Kenya.<br><br>(Qualitative with case study)	13 oncology nurses<br><br>At the hospital	The researchers interviewed participants and identified three main aspects in the management of cancer pain: how they assist patients in managing their pain themselves, the types of interventions used to support self-management of cancer pain, and the challenges faced by patients.
23	Jadmiko, et al (2021)<br><br>Indonesia	Provides a new insight into in-depth information about cancer patients' experiences with self-management of pain.<br><br>(Qualitative (Phenomenologi study))	8 cancer patients<br>At the hospital	There are four themes that depict participants' experiences in their efforts to self-manage pain, namely by: (1) Engaging in daily activities, (2) Engaging in religious activities, (3) Social interactions, and (4) Seeking pleasant environments.

Table 3. Integration of education with pain self-management

The form of integration	Methods and Mediums	Author
Implementing interventions through direct face-to-face methods (at the hospital or home visits) and via online means (telephone, video conference, eHealth, Microsoft Teams, Zoom, or WhatsApp).	Approach: Individual and Group<br><br>Media: Telephone, video conference, eHealth, Microsoft Teams, Zoom, or WhatsApp Booklet, Pain management modules, pamphlets, diary	1,2,3,4,5,6,7,8, 10,11,16,19,20,22
Creating models, guidebooks, and questionnaires	Approach: Individual and Group<br><br>Media: Pain management modules, pamphlets, diary	9,15,17,18,23
Developing and modifying tailored pain self-management programs or interventions for cancer patients/types of cancer.	Approach: Individual and Group<br><br>Media: Health education booklet	12
Non-pharmacological pain management : cognitive behavioural therapy	Approach: Individual<br><br>Media: Pain management modules, pamphlets, diary	13,14,21
